# Point-of-Care Diagnostics in Low Resource Settings: Present Status and Future Role of Microfluidics

**DOI:** 10.3390/bios5030577

**Published:** 2015-08-13

**Authors:** Shikha Sharma, Julia Zapatero-Rodríguez, Pedro Estrela, Richard O’Kennedy

**Affiliations:** 1 Biomedical Diagnostics Institute (BDI), Dublin City University, Glasnevin, Dublin 9, Ireland; E-Mails: shikha.sharma@dcu.ie (S.S.); julia.zapatero@dcu.ie (J.Z.-R.); 2 School of Biotechnology, Dublin City University, Glasnevin, Dublin 9, Ireland; 3 Department of Electronic and Electrical Engineering, University of Bath, Bath BA2 7AY, UK; E-Mail: P.Estrela@bath.ac.uk

**Keywords:** low-cost biosensors, point-of-care diagnostics, lateral flow immunoassay, microfluidics, global health

## Abstract

The inability to diagnose numerous diseases rapidly is a significant cause of the disparity of deaths resulting from both communicable and non-communicable diseases in the developing world in comparison to the developed world. Existing diagnostic instrumentation usually requires sophisticated infrastructure, stable electrical power, expensive reagents, long assay times, and highly trained personnel which is not often available in limited resource settings. This review will critically survey and analyse the current lateral flow-based point-of-care (POC) technologies, which have made a major impact on diagnostic testing in developing countries over the last 50 years. The future of POC technologies including the applications of microfluidics, which allows miniaturisation and integration of complex functions that facilitate their usage in limited resource settings, is discussed The advantages offered by such systems, including low cost, ruggedness and the capacity to generate accurate and reliable results rapidly, are well suited to the clinical and social settings of the developing world.

## 1. Introduction

The long-term social and economic stability of a country is highly dependent on improved health. The worldwide advancement in health and technology in the last 50 years is far greater than in the previous 500 years [1]. In spite of technological advancements, developing countries still struggle with numerous health challenges. In addition to the non-communicable diseases common to all countries, such as obesity, diabetes, and cancer, developing countries still struggle with an additional disease burden from diseases which are preventable or treatable in the developed countries [2,3]. Though the burden of diseases is highest in the developing countries, laboratory or medical testing facilities are often limited and inaccessible to most patients, resulting in high mortality rates. The fundamental basis of evidence-based treatment is to find the cause of a patient’s illness. Reliable and accurate diagnostic investigations play a vital role in healthcare decisions, choice of treatment, and achievable survival. Although more than 50% of treatment decision-making is based on some diagnostics, it still accounts for less than 3% of the cost of healthcare. Unfortunately, a major aspect in developing countries is that they often lack modern laboratories, fully automated instruments that provide highly reproducible, quantitative, and hence sensitive and accurate diagnostic results. Rural areas often lack access to even basic diagnostic devices and trained personal. The scarcity of running water and reliable electrical services are additional challenges for delivering healthcare in these areas [4]. In order to circumvent these issues the emergence of rapid and easy-to-use point-of-care (POC) tests can dramatically enhance a physician’s ability to diagnose patients’ diseases rapidly and accurately.

POC testing is a necessary component of affordable worldwide healthcare allowing rapid testing at, or near, the site of patient care. In limited-resource or non-existent healthcare settings, or where it is very hard to physically access relevant facilities, POC approaches can save hundreds of thousands of lives every year [5]. The global market for point-of-care diagnostics is expected to reach $27.5 billion by 2018 at an estimated compound annual growth rate (CAGR) of 9.3% from 2013–2018 [6]. The potential benefits of these rapid or POC diagnostics include the short turnaround time (TAT), minimal manual input, portability, low cost, immediate clinical decision-making, and “ease-of-use” formats so that they can be successfully deployed at the primary care level and are particularly amenable for use in remote settings with poor or no laboratory infrastructure [7]. In this paper, we review the diseases involved and the current healthcare challenges encountered by developing countries. We will discuss the present status of POC diagnostics in the developing world, highlighting, in particular, lateral flow technology-based devices. The latter platforms can make a major impact on diagnosis and treatment in limited-resource healthcare as they are low-cost, easy to transport, and use and generate results rapidly. Furthermore, we provide an overview on the latest advancements in the field of microfluidics-based devices, which allow miniaturisation of complex fluid handling steps, ease of multiplexing, and have integrated detection systems.

### Disease Burdens in the Developing World

At present, the global population is more than 7 billion with a growth rate of around 1.14% per year, and it is expected to reach 9 billion or above by 2050. Developing countries account for 84% of the global population, with Asia accounting for approximately 60%, contributed to mainly by the two most populous countries, China and India [8]. In spite of accounting for 90% of the global disease burden, the developing countries account for only 12% of global health expenditure [9]. The occurrence of both communicable and non-communicable diseases, such as diabetes, obesity, cardiovascular, and cancer, *etc.*, is increasing at a much higher rate in developing countries than in the developed countries. Every year, acute respiratory infections account for approximately 4.3 million deaths, 1 million people die from malaria, and about 5 million from AIDS and tuberculosis in the developing world [10]. In 2013, approximately 8 million people died with non-communicable diseases before their sixtieth birthday in these countries [11]. Developing countries are still struggling to meet the challenges of those diseases that are preventable or treatable in the developed world.

Access to diagnostic tests and quality community screening can dramatically improve public health in the developing world. Accurate diagnosis plays a crucial role in identifying the incidence and root cause of diseases at both the individual and population levels. Most of the commercially available diagnostic tools for diseases are insufficient in meeting the needs of the limited-resource setting or a weak infrastructure-based healthcare set-up. These shortcomings include long TATs for testing with associated delay in availability of data resulting in late decision making. The development of point-of-care (POC) tests for limited-resource settings is an active and promising area for improving disease management. In addition to the increase in private-sector investment in diagnostics development, a number of initiatives funded by the public sector, including the European Union’s Seventh Framework Programme (FP7) and Horizon 2020, the UK’s Department for International Development, the Foundation for Innovative New Diagnostics, the U.S. Global Health Initiative, and the Bill & Melinda Gates Foundation are currently running with the objective to develop new diagnostic tools to support the healthcare systems of the developing world. An ideal diagnostic test for a limited-resource setting should have the following characteristics: be user friendly; generate results rapidly, allowing medical personnel to diagnose disease and start immediate treatment; be robust (no requirement for refrigerated storage); avoid requirements for complex pre-processing of biological samples; be portable, inexpensive, and highly sensitive; and have the requisite specificity [12]. Lateral flow devices are among the most established point-of-care testing platforms that have been successfully applied in areas where timely medical care is a challenge and they have most of the requisite characteristics previously described [13]. Numerous lateral flow test strips have been developed for diagnosis of infectious diseases as well as a range of disease-associated biomarkers, e.g., in cardiac disease and cancer.

## 2. Lateral Flow Tests

Lateral flow or Lateral Flow Immunoassay (LFIA) technology is the most simple and successful rapid diagnostic testing platform derived from the latex agglutination test developed by Singer and Plotz in 1956 [14]. Since its introduction, the technology and its applications in various fields, has continued to evolve. Its remarkable global growth can be attributed to numerous factors including the need for early stage detection of diseases like cancer, continuous demand for screening tools for preventive management of infectious diseases, and the requirement for cost-effective devices with little or no maintenance requirements.

### 2.1. Impact on Developing Countries

The human pregnancy test was the key application driving the initial development of a rapid test platform; this made great strides in the 1970s in decentralized testing. Initially, the rapid tests for infectious diseases, including tests for human immunodeficiency virus (HIV-1 and HIV-2), TB, and hepatitis B were introduced in a dipstick format in developing countries. In the late 1980s, the first immunochromatographic strip (ICS) lateral flow format was introduced for disease diagnosis. Since then the technology, its applications, and the associated industry have continued to evolve. These test platforms have attractive performance features for developing countries, including stability for more than one year, precise performance, interpretation by minimally trained users, usability with a wide range of specimens, and no refrigeration required during shipping and storage. There are several thousand untrained persons performing rapid testing in developing countries [15]. Globally, more than 100 companies are producing a wide range of lateral flow tests. Table 1 shows some of the commercially available LFIA strips and their performances. The data is divided into three different categories: infectious diseases, cancer, and cardiac diseases. In 2010 the US market accounted for US$1,680 million (50%), the European market around 40% (US$1,344 million), and the rest of the world accounted for 10% (US$336 million) of the total rapid test market [16].

The development of LFIA tests for use in developing countries has focused mainly on infectious diseases, including HIV, Strep A/B, legionella, malaria, respiratory viruses, influenza, meningitis, filariasis, tuberculosis, measles, mumps, rubella, chlamydia, and gonorrhea [17]. The market leader for these tests, Alere, covered approximately 35% of the market. Various studies have shown that early diagnosis using LFIA platforms has benefits in disease prevention and treatment and also lowers the risk of transmission. LFIA for HIV testing has expanded rapidly worldwide [18]. The World Health Organization (WHO)/UNAIDS estimates that more than 5 million people received antiretroviral therapy, and more than 29 million people were tested for HIV infection in developing countries in 2010 [19]. The HIV LFIA testing has also resulted in increasing the proportion of pregnant women who received their test result within a specified timeline and initiated antiretroviral therapy. The Ora Quick Rapid HIV-1/2 Antibody Test is a quantitative test for rapid HIV monitoring based on lateral flow technology for oral fluid specimens even at low concentrations of HIV antigens in oral fluid. The additional LFIA-based testing studies includes C-reactive protein determination among neonates suspected of having meningitis in South Africa, a tetanus immunity test in Iran, a test for trypanosomiasis in Angola, and a test for visceral leishmaniasis in Brazil, India, Kenya, and Sudan [12]. An early diagnosis using LFIA-based tests for pulmonary tuberculosis has also reduced mortality and transmission in low-resource settings. Cryptococcal antigen and *Plasmodium falciparum* malaria testing based on LFIA has also shown promising sensitivity for detection of disease and has benefited the developing countries in terms of reduced mortality [20,21].

**Table 1:** Overview of commercially available lateral flow test strips: For detection of infectious diseases, cancer, and cardiac diseases.

	Company	Product Name	Disease	Analyte/Antigen (Ag)	Required Sample	Detection Time (Min)	Sensitivity	Specificity
**Infectious Diseases**	Alere	Binax NOW Filariasis	Lymphatic filariasis	*Wuchereria bancrofti*	100 µL WB/S/P	10	-	-
	IMMY	CrAg A	Cryptococcal meningitis	*C. neoformans C. gattii*	40 µL of S/CSF	10	100%	94%
	Alere	Binax NOW	Malaria	*Plasmodium Ag*	15 µL of WB	15	*P. falciparum*: 99.7%, *P. vivax*: 93.5%	*P. falciparum*: 94.2%, *P. vivax:* 99.8%
	Quidel Corp.	Quick Vue RSV Test	Infantile bronchiolitis	Respiratory syncytial virus (RSV) Ag	Nasal swab, aspirate and wash	15	92%-swab; 99%-aspirate; 83%-wash	92%-swab; 92%-aspirate; 90%-wash
	Alere	Alere Determine HIV-1/2 Ag/Ab Combo	AIDS	HIV-1/2 antibodies and free HIV-1 p24 Ag	50 µL of WB/S/P	20	-	99.75%
	Alere	Panbio Dengue Duo Cassette	Dengue fever	IgM and elevated IgG	WB/S/P	15	85.50%	91.60%
	Alere	Alere™ Influenza A & B Test	Influenza	Influenza A and B nucleoprotein Ag	Nasal swab	10	Flu A 93.8%; Flu B 77.4%	Flu A 95.8%; Flu B 98%
	Alere	Alere Determine TB LAM	Tuberculosis (TB) in HIV positive patients	Lipoarabinomannan (LAM) Ag	60 µL of Urine	25	51.7% for <100 CD4 cell count	>98%
	Trinity Biotech	Uni-Gold™ Legionella Urinary Antigen PLUS	Legionnaire’s Disease	Legionella pneumophila serogroup 1 Ag	150–200 µL of Urine	15	82.1%	99.2%
	Alere	Binax NOW	Pneumonia	*S. pneumoniae* Ag	Urine/CSF	15	Urine-86%, CSF-97%	Urine-94%, CSF-99%
**Cancer**	CTK Biotech	On Site PSA Rapid Test	Prostate cancer	Prostate specific antigen (PSA)	60–90 µL of S/P	10	Relative: 100%	Relative: 99%
	Alere	Alere NMP22 BladderChek	Bladder cancer	Nuclear matrix protein (NMP22)	4 drops of Urine	30	99% when combined with cystoscopy	99% NPV along with cystoscopy
	Alere	Clearview iFOBT	Colon cancer	Faecal Occult Blood	Faeces	5	93.60%	99.10%
	Arbor Vita Corp.	OncoE6 Cervical Test	Cervical cancer	E6 oncoproteins	Cervical swab	150	84.6%	98.5%
	Quicking Biotech Co., Ltd	CA125 rapid test kit	Ovarian Cancer	CA125 Ag	100 µL of S	10	-	-
	Innovation Biotech	AFP Test	Hepatocellular Cancer	Alpha fetoprotein Ag	S/P	10	25 ng/mL	99%
**Cardiac Diseases**	LifeSign	StatusFirst CHF NT-proBNP	Congestive Heart Failure	NT-proBNP	3 drops of P	15	20 pg/mL	-
	BTNX Inc.	Rapid Response CK-MB Test	Myocardial infarction (MI)	Creatine kinase MB (CKMB)	WB/S/P	10	5 ng/mL	99.80%
	Boditech Med Inc.	ichroma™ CK-MB Test	Myocardial infarction (MI)	Creatine kinase MB (CKMB)	75 µL of WB/S/P	12	3 ng/mL	-
	Response Biomedical Corp.	RAMP MYOGLOBIN TEST	Acute myocardial infarction (AMI)	Myoglobin	WB	10	2.36 ng/mL	-
	Trinity Biotech	Meritas^®^ Troponin I	Myocardial infarction (MI)	Troponin I	200 µL of WB/P	15	0.036 ng/mL	-
	BTNX Inc.	RAPID RESPONSE D-DIMER TEST	Venous Thromboembolism (VTE)	D-Dimer	WB/P	10	500 ng/mL	-
	American Screening Corp.	Instant-View Troponin I	Acute myocardial infarction (AMI)	Troponin I	200 µL of WB/S	10–20	-	-

WB-Whole blood, S-Serum, P-Plasma, CSF-Cerebrospinal fluid.

LFIA platforms have also impacted the cardiac disease diagnosis segment. The rapid results using LFIA platforms in cardiac ischaemia have resulted in the rapid diagnosis and precise medical treatment given at right time ensuring reinstatement of the blood flow to the heart. Using LFIA-combined with cardiac markers (CK-MB, myoglobin and troponin I/T, D-Dimer, hsCRP, and BNP (B-type natriuretic peptide)) has improved the early diagnosis of ischaemia resulting in better patient management [22]. For example, in a study involving 817 patients enrolled in the ED for suspected AMI, the median time from sampling to reporting of results was 71.0 min for the central laboratory testing of CK-MB (by using Abbott’s AxSYM assays), whereas the POC testing of CK-MB, myoglobin, and cTnI using LFIA based test is 15–20 min [23]. The leader and innovator in the cardiac marker POC segment is Alere. There are many other companies that sell LFIA tests for all or one of these cardiac marker panels. These are listed in Table 1.

### 2.2. Principle of a Lateral Flow Immunoassay

Lateral flow immunoassay (LFIA) is based on the use of a nitrocellulose, polymer, paper, or other composite substrate membrane which facilitates the separation, capture, and detection of the target analyte(s) of interest. The various components of a LFIA have the capacity to transport fluid, including blood or serum, under capillary action, and thus no external pumps are required.

**Figure 1:**
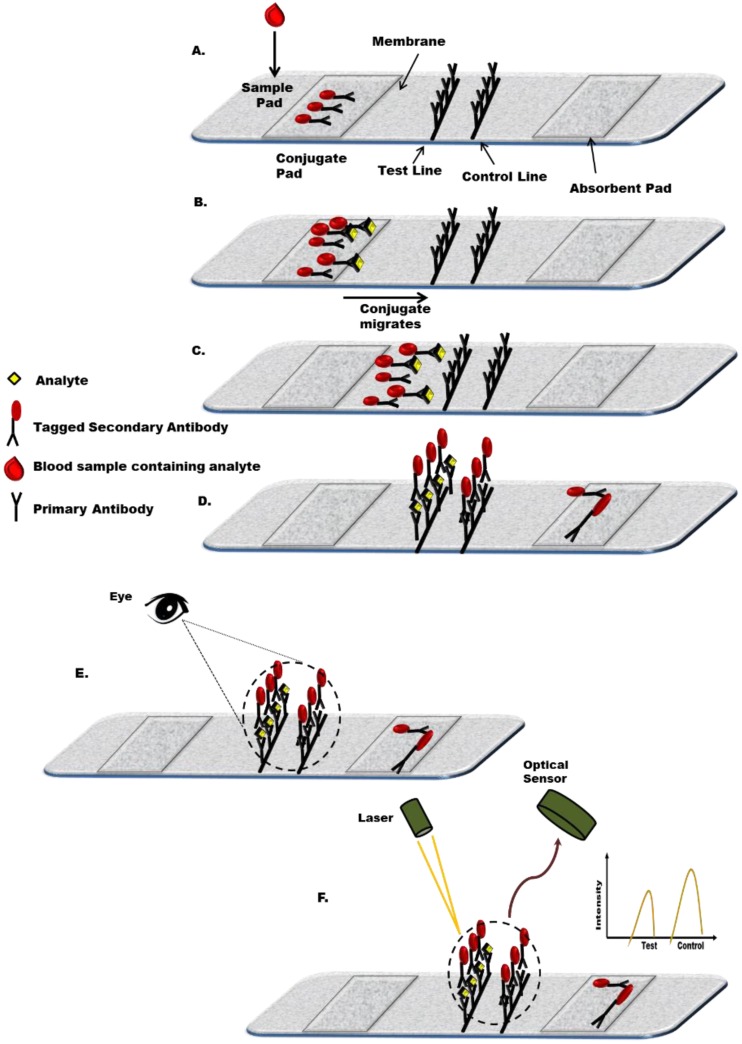
Schematic representation of the principle of sandwich format of a lateral flow immunoassay. (**A**) Labelled lateral flow test strip; (**B**) Migration of sample from sample pad to conjugate pad; if the desired antigen is present in the sample the conjugate will bind to it creating a complex (antigen-antibody-label); (**C**) The complex flows through the membrane towards the test line; (**D**) Immobilised Ab captures the complex and free labelled Ab and forms test and control lines, respectively; (**E**) Qualitative results-visual detection; (**F**) Quantitative detection, e.g., optical detection (depending on label used).

There are various formats for lateral flow assays. In a simple capture format (Figure 1) the target analyte from the sample interacts with a labelled antibody, already pre-loaded on the strip, and migrates up the strip until it encounters another target-specific antibody, which is immobilised on the strip. The capture antibody-antigen-labelled antibody is then evident as a line which can be seen by eye or can be measured using a detector. A control is also present which uses a non-specific antibody and the presence of the associated line clearly demonstrates that the assay is working correctly. Other formats, e.g., competitive assays, are also possible, and may be particularly relevant where the target analyte does not possess multiple epitopes for antibody binding [17].

**Figure 2:**
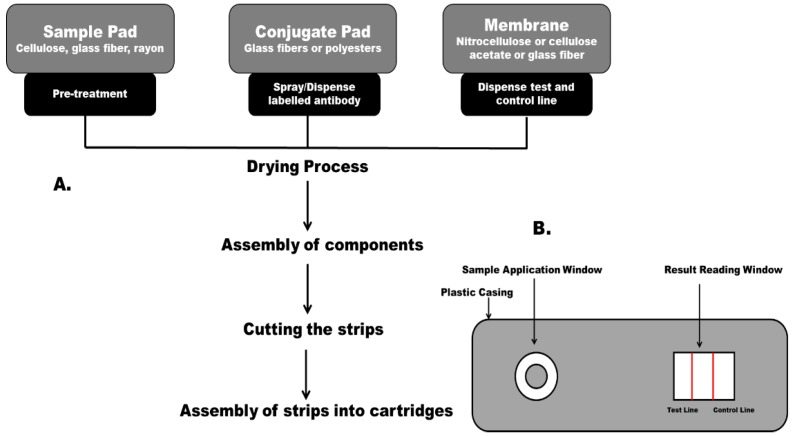
(**A**) Outline of manufacturing process for lateral flow tests; (**B**) Top view of a lateral flow immunoassay test cartridge.

In practice, the system works as follows. Briefly, a sample is introduced on to the sample pad through the sample application window present on the plastic casing, as shown in Figure 2. The sample migrates through the sample pad to the conjugate pad, where the recognition element (antigen/antibody) is already available in a dried form. Here, the target analyte interacts with the dried labelled recognition element and the resulting complex further migrates to the next segment of the LFIA. This segment, the reaction matrix, consists of a hydrophobic nitrocellulose or cellulose acetate membrane where anti-target antibodies or antigen have been immobilised (depending on the type of format) in a line or band format across the membrane to act as a capture zone for capturing the labelled conjugate. This is followed by the presence of another zone, the control zone, which checks for assay functionality. Superfluous reagents, buffers, and assay fluids further migrate toward the absorbent pad and are absorbed as waste. The results are interpreted either by the naked eye or using a reader which evaluates the presence or absence of a test line along with the control line [24]. Figure 1 shows a schematic representation of the principle of a sandwich format lateral flow immunoassay. LFIA-based strips have different detection formats: sandwich and competitive. Figure 2 outlines key stages involved in a manufacturing process for lateral flow test strips.

The different constituents of a lateral flow test and the raw materials used for each constituent that play a vital role in the quality of the results generated are critically assessed in the next sections.

#### 2.2.1. Biorecognition Components

The high specificity and selectivity of the biorecognition element are key for the performance of the assay. There are many different recognition elements that can be employed but antibodies are by far the most common and well established. Antibodies used in LFIA can be monoclonal, polyclonal, or both together, but increasingly recombinant antibodies are also being used. Traditionally polyclonal and monoclonal antibodies have been used extensively. Both have their own advantages, including the high specificity of monoclonal antibodies, which helps in decreasing non-specificity and cross-reactivity, while the ability of polyclonal antibodies to recognize multiple epitopes on a single antigen, as well as their low cost production in bulk quantities, are valuable properties. Many rapid tests employ 2–3 types of antibodies, e.g., for binding specifically to the target antigen, for carrying the label for visualisation, and for acting as a control. The stationary phase includes antibodies immobilised at the capture line location (specific to target analyte) to capture antigen, and another at the control line. The mobile phase contains the antibody bound with a label, e.g., enzymes, fluorescent tags, gold nanoparticles, quantum dots, or latex beads, which first interact with the target analyte in a direct or sandwich immunoassay format and then come in contact with the immobilised capture antibodies. Recently, the potential of using recombinant antibodies is being explored. This derives from the fact that their specificity can be carefully tuned to allow either broad or highly specific binding, allowing the analysis of groups of related targets or analysis of an individual target. They can also be functionalised to allow high levels of immobilisation with optimal orientation. In addition, the labels may be molecularly linked to the antibody, e.g., use of green or other fluorescent proteins, thus avoiding labelling steps which may lead to conjugations that may reduce or even inhibit the antigen- binding capacity of some antibody molecules. Issues related to stability may be overcome by additional engineering to create formats e.g., Fabs or scAbs, that work well in LFIA. In addition to antibodies, aptamers, affimers, more complex molecularly imprinted polymers, and several other binders are being explored as promising alternative capture probes [25,26,27]. There are three types of aptamers: DNA, RNA, and peptide aptamers. Single-stranded DNA or RNA (ssDNA or ssRNA) molecules can bind to selected targets, including proteins and peptides, with high affinity and specificity. Organic molecules having molecular weights in the range of 100–10,000 Da are ideal targets for aptamers.

#### 2.2.2. Sample Application Pad

Cellulose, glass fibre, rayon, or cross-linked silica are common materials used to make sample application pads. The major role of the sample pad is to allow continuously uniform migration of the sample to the conjugate pad whilst making the sample compatible with the other components of the test system. This is usually achieved by pre-treatment of the sample by separating the plasma or serum from whole blood and may involve trapping of erythrocytes and other cells, adjustment of pH, and elimination of interferents, in order to allow efficient interaction of the target analyte with antibodies and conjugates.

#### 2.2.3. Conjugate Pad

The sample migrates from the sample pad to the conjugate pad where it mixes with the dried conjugates (e.g., antibodies specific to target analyte tagged with a label). Conjugate pads are usually made up of glass fibres or polyesters and are often pre-treated with different proteins, polymers, and surfactants to make them hydrophilic in order to ensure stability and optimum release of the conjugate. The material composing a conjugate pad often plays a vital role in determining the overall shelf-life of the LFIA test by ensuring the stability of antibodies, labels, and the associated conjugates, and allowing release of the conjugates from the pad [17]. During manufacture, the conjugates are dispensed on the pad using a quantitative dispenser such as the BioDot AirJet Quanti 3000 in order to maintain the uniform dispensing and, thus, the reproducibility of the components. High levels of control and precise dispensing of reagents are vital for good performance. Variations in conjugate mixing, deposition, or release from the pad result in significant deviations in assay performance.

#### 2.2.4. Membrane

A wide variety of membrane types and pore sizes are commercially available. The selection of the membrane depends on the analyte and the application of the test. Typically, a hydrophobic nitrocellulose (high protein binding), glass fibre (non-protein binding), or cellulose acetate (low protein binding) membranes are used for LFIA. Nitrocellulose membranes are the most widely accepted materials that have been efficaciously applied for LFIA tests due to their distinctive characteristics including high affinity for proteins, ease of wetting, lack of interference with assay, relatively low cost, commercial availability of several versions of nitrocellulose, true capillary flow characteristics, and comparative ease of handling. The reaction area is comprised of two lines or zones on the nitrocellulose membrane, known as the test (or capture) and control lines. The capture line consists of specific antigen or antibodies immobilised to capture the target analyte whereas the presence of the control line confirms the proper flow of the liquid or performance of the assay. Several factors including accurate dispensing of biological components and proper blocking and drying play a key role in refining sensitivity of the assay.

#### 2.2.5. Absorbent Pad

The absorbent pad is usually made up of high-density cellulose and is placed at the end of the strip or after reaction region. The main role of an absorbent pad is to maintain the flow of reagents over the membrane and to avoid false positives by not allowing back flow of reagents. It is also called a wick or the sink in an LFIA system.

#### 2.2.6. Backing Materials for the Membrane

In order to facilitate easy handling and to provide strength to the fragile membranes used in the LFIA system, all capillary beds are usually mounted on a backing material. The materials used for providing the requisite rigidity are typically polystyrene or different variants of plastic which are coated with a pressure-sensitive adhesive to hold all the components together in a defined format. The compatibility of proteins with adhesives over a long period of time plays an important role in maintaining the prescribed shelf-life of the product. The backing serves as a structural support to the LFIA system to which several layers (e.g., pads) performing different functions are laminated and the entire entity is housed in a single cassette.

#### 2.2.7. Detection Label

The choice of a label used for detection normally depends on the application of the LFIA system. Labels such as latex beads, colloidal carbon, colloidal gold, fluorescent tags, enzymes, streptavidin, or gold nanoparticles, are conjugated with an antibody or an antigen depending on the format of the LFIA system [28,29,30]. Several forms of latex beads coupled with coloured dyes, fluorescent dyes, or magnetic elements are commercially available and can be attached covalently or electrostatically with the proteins. The presence of coloured labels avoids the requirement for costly detectors and allows the visual detection of the target analyte. The capacity for coupling latex beads with different colours can allow multiple analyte detection on a single test membrane. Evolution of nanomaterials has improved the sensitivity of these assays tremendously. The inherent properties of gold nanoparticles, including strong optical properties (intense colour), and the smaller size of gold particles (20–40 nm) in comparison to latex beads (100–300 nm) make them good candidates for enhancing sensitivity and, thus, for application in LFIA systems. The colour intensity of gold nanoparticles is much higher than coloured latex particles, thus providing better discrimination of low level positives in an assay. Several other nanomaterials, including silver ions on gold nanoparticles, have been integrated into LFIAs to further enhance the sensitivity of the system. In the ideal situation, the assay may be able to detect multiple analytes. This can be achieved by running multiple lanes on the one strip in parallel with individual detection sites. Alternatively, barcoding is possible with different labels demonstrating the presence of different analytes. Many different formats are possible but in resource-limited environments simplicity in use, sensitivity, and specificity are key.

#### 2.2.8. Detection System

The majority of the lateral flow assays use visual detection. The incorporation of gold nanoparticles or latex beads as labels allows visual examination of colours at the test and control lines, resulting in qualitative or semi-quantitative analysis. The generation of rapid results in critical or intensive care unit (ICU) patients is of great assistance to doctors to make an instantaneous decision and initiate treatment. The use of colloidal gold, paramagnetic particles, and fluorescent dyes as labels in lateral flow formats has opened up new horizons for LFIA technology by allowing true quantitative testing. The development of quantitative assays in lateral flow formats requires an additional fluorescence or optical strip reader which allows measurement of the intensity produced at the test and control lines of the strip [31,32,33]. The readers are based on the use of different wavelengths of light for illumination, in combination with a camera or charge-coupled device (CCD) imaging technology which allows capture of images of test and control lines. Furthermore, an integrated image processing algorithm converts the intensity of the lines to the corresponding analyte concentration. The selection of detector is chiefly determined by the detection label used in analysis. Fluorescent or paramagnetic particles allow the achievement of 1–2 orders of magnitude of higher sensitivity in comparison to visual labels, such as colloidal gold or coloured latex. The precise positioning of the test and control lines on the strip and background correction is of crucial importance for optimal performance in these systems. More recently the use of mobile phone-based detection strategies is being explored and this may well provide a valuable strategy for use in “resource-poor” environments.

### 2.3. Limitations of “Traditional” POC Approaches

As discussed above, LFIA techniques have allowed quick and simple point-of-care diagnosis of a variety of diseases in different environments. However, their simplicity limits their performance and in many cases more complex devices are necessary for accurate diagnosis. LFIA or immunochromatographic (ICS)-based strips have successfully been implemented for the diagnosis of several transferable diseases such as HIV, tuberculosis, and sexually-transmitted diseases (STDs) in low-resource settings. ICS strips rely on capillary forces to control fluid flow. By the nature of sample addition to such devices, there may be some variations in the amount of sample added initially and actually used in the test and this can result in poor precision. In addition, in many test formats pre-treatment of samples becomes mandatory in cases where the sample matrix is not a fluid or where significant interferents are present. In addition to this, restrictions in the limit of detection of these platforms may restrict their use to the determination of analytes that are highly abundant in the sample tested, such as lymphocytes in HIV tests or pathogenic microorganisms in malaria tests. Hence, the results may be only qualitative or semi-quantitative, even when confirmed with a reader. The large coefficient of variation (CV) associated with an LFIA test also limits the test reproducibility and, therefore, its ability to confirm the presence of the disease [17]. Variable test performance due to variations in environmental factors such as temperature, humidity, heat, air, and sunlight are added complications [34].

## 3. The Future: Microfluidics as an Emerging Platform for Point-of-Care Diagnosis

Over the last two decades, microfluidic technologies have experienced a significant growth in applications in the field of diagnosis. Microfluidics-based instrumentation has shown promising results in several of the main laboratory techniques, including blood chemistries, immunoassays, nucleic-acid amplification tests, and flow cytometry [35]. Microfluidics allows the miniaturisation and automation of diagnostic devices and has been touted as capable of supplanting (or at least complementing) LFIA devices for health care applications in POC settings. It presents several advantages compared with LFIA technologies. These include the ability for extensive use of complex multiplexing approaches and formats, the capacity for inclusion of sequential sample pre-conditioning steps of different types, reagent storage, the use of multiple steps involving reagent addition, mixing and washing, the potential for incorporation of centrifugal steps at various speeds, and the application of a range of detection strategies leading to greater sensitivity and clarity of results.

### 3.1. Microfluidic Devices in Developing Countries

Fully automated instruments located in centralised laboratories are capable of providing highly reproducible, quantitative, and sensitive results. Unfortunately, in developing countries, the necessary infrastructure for these modern laboratories, if available, is often only present in the major cities’ hospitals or in regional centres. Rural areas often lack access to the requisite technology and the necessary trained personal to perform the tests and carry out the subsequent diagnoses, especially where the situation is complicated for patients with multiple concomitant infections and malnutrition. When developing a diagnostic device intended for use in resource-poor areas, it is of utmost importance to ensure it conforms to the stringent performance requirements previously listed in earlier sections of this review. Microfluidic-based systems are able to fulfil these requirements by miniaturising and automating complex diagnostic procedures into a portable device [35].

**Figure 3:**
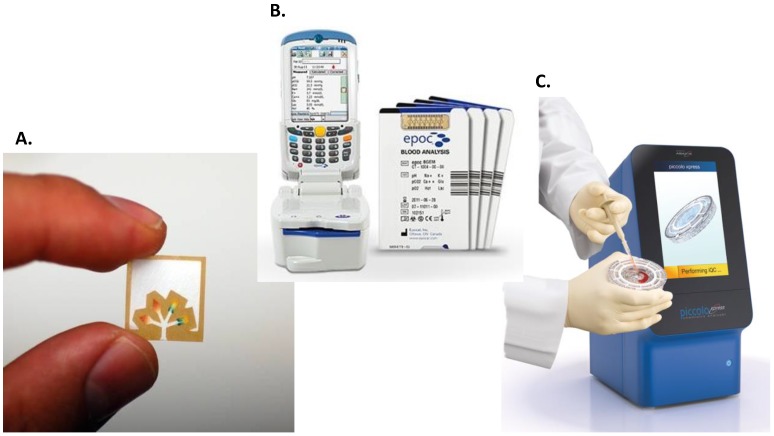
Examples of microfluidic-based platforms. (**A**) Paper-based microfluidic device for simultaneous quantification of glucose and other analyte in urine. Taken from http://gmwgroup.harvard.edu with permission from Prof. Whitesides; (**B**) Lab-on-a-chip platform developed by Alere for blood chemistry analysis. Reproduced with permission from Alere; (**C**) Lab-on-a-disc diagnostic device designed by Abaxis. Reproduced with permission from Abaxis.

Several academic and industrial groups have explored the potential of microfluidic-based technologies to be used in resource-poor settings in developing countries. Patterned-paper technology (Figure 3) is being widely evaluated since the Diagnostics for All’s (DFA) mission was founded in 2007 by George Whitesides and his group in Harvard University aiming to improve diagnostics approaches in developing countries. DFA is working on the development of low-cost, easy-to-use POC tests that require no power or trained clinicians [36]. Whitesides’s group demonstrated the potential of a patterned paper device for monitoring drug-induced hepatoxicity in at-risk individuals, such as those being treated for tuberculosis and/or HIV. The test linearity showed that aspartate aminotransferase and alanine aminotransferase (indicators of liver function) assays were linear across the clinical range (40–200 U/L), and CV values were <10% for both tests [37]. Other microfluidic platforms such as lab-on-a-disc and lab-on-a-chip devices are being presented as attractive solutions for POC settings (Figure 3). Hugo *et al.* [38] analysed the implementation of a centrifugal microfluidic platform for POC applications in South Africa. The results of the microfluidic disc example showed how functions such as valving, mixing, pumping, and separation of fluids can be integrated into the centrifugal microfluidic platform for diagnostic applications. Work has also begun on the development of a lab-on-a-chip for malaria diagnosis consisting of a disposable disc and a portable RT-PCR machine. A third party evaluation of the prototype device yielded a sensitivity of 96.7% and a specificity of 100% (*n* = 38) [39]. The use of a microfluidic platform for diagnostic of the *Mycobacterium tuberculosis* has also been reported. Jing *et al.* [40] integrated an on-chip airborne bacteria capture system and a rapid bacteriological immunoassay into an automated microfluidic system that was able to detect the pathogen in 50 min, eliminating the need of a culturing step. Other researchers have introduced a magnetic nanoparticle/quantum dot-based immunoassay for the detection of pathogens responsible for waterborne and foodborne diseases (e.g., *E. coli* 0157:H7, Salmonella, *etc.*) using a microfluidic chip. The chip consists of two microchannels embedded with magnets, where immunomagnetic separation of the bacteria takes place. Detection is achieved by labelling the captured bacteria with bioconjugated quantum dots which fluorescence [41]. To date, however, these microfluidic devices are still not suitable to use in the developing world, and most of them remain in a proof-of-concept state, lacking the clinical trials needed to clear the test. Some companies have, nevertheless, managed to satisfy all the criteria needed to launch their POC tests on the market. Table 2, adapted from Chin *et al.* [7], shows a compilation of the FDA-approved microfluidics-based technologies for “on-site” tests.

**Table 2:** List of FDA-approved microfluidic-based POC tests. Adapted from Chin *et al.* [7].

Company	Test Name	Tested Analyte/Parameter	Sample Required	Components	Training Requirements and Other Characteristics	Results Type and Turnaround Time
Abaxis	The Piccolo^®^	Blood chemistries	100 µL of WB, S or P	Disposable test discs and portable analyser.	No operator intervention is required.	Quantitative results in about 12 min.
Biosite (Alere)	Alere Triage^®^ MeterPro	Blood/urine chemistries including “waived” Lipid and liver panels	225 µL of WB, P or urine	Disposable cartridges, and meter.	No training required. Built-in quality controls.	Qualitative results in 15–20 min.
Epocal (Alere)	The epoc^®^ Blood Analysis System	Blood chemistries	92 μL of WB	Test cards and wireless card reader with a host mobile computer.	No training required. RT- test card storage. Automated, built-in QC/QA calibration and quality tests	Quantitative results in about 30 s.
Focus Dx (Quest)	Simplexa	Flu A/B & RSV	Nasopharyngeal swabs	PCR platform (3M™ Integrated Cycler) and amplification discs with sample wells.	Used by physician.	Quantitative results in 1 h.
HandlyLab (BD)	BD MAXTM GBS Assay IDI-Strep B Assay	Group B Streptococcus (GBS)	Vaginal/rectal swab samples	Cepheid Smart Cycler^®^ RT-PCR system, disposable cartridge and computer system.	Trained personal required. Can be performed in moderate complexity laboratories.	Qualitative results in 1 h.
i-STAT Corp (Abbot)	i-STAT Analyser	Blood chemistries; coagulation; cardiac markers	17–95 μL of WB, depending on cartridge type	Handheld analyser and self-contained cartridges.	Training is required. Portable. Cartridges can be stored at room temperature.	Quantitative results in less than 15 min.
Idaho Technologies	FilmArray RP	Panel of respiratory pathogens	Nasopharyngeal swabs	FilmArray instrument and freeze-dried reagents in pouches	No precise measuring or pipetting required.	Qualitative results. 2 min. of hands-on time; about a 1-h turnaround time.
IQuum	Liat™ Influenza A/B Assay	H1N1 influenza viral RNA	Nasopharyngeal swab samples	Benchtop analyzer disposable assay tubes	Use under CLIA requirements. Can be operated by minimally trained users.	Qualitative results in less than 30 min.
Micronics (Sony)	ABORhCard	ABO and Rh blood typing	50 μL of fingerstick WB	Disposable cards that with anti-A, B and D antibodies printed into discrete microfluidic channels.	No training required. No refrigeration needed. Long shelf-life.	Qualitative results in approximately 2 min.
Sphere Medical	Proxima	Blood chemistry	Arterial WB	Disposable multi-parameter microanalyser, silicon chips.	Use by trained clinician.	Quantitative results in approximately 3 min.
TearLab	TearLab Osmolarity System	Osmolarity of human tears (diagnosis of Dry Eye Disease)	50 nL of tear film	Disposable microchip works in conjunction with a pen that convey data to the osmolarity reader.	Professional *in vitro* diagnostic use only.	Quantitative results in seconds.

WB-Whole blood, S-Serum, P-Plasma.

The Alere epoc^®^ blood analysis system (see Figure 3) is a POC platform for blood gas, electrolyte, and metabolite testing. This system integrates a variety of assays that run simultaneously in a disposable and low-cost card, producing reliable results determined on a reusable wireless reader. Alere developed the Pima CD4 test (CE marked), consisting of disposable cartridges and a benchtop cytometer. In this test, the dried reagents are sealed in the cartridge, which does not require refrigeration, and the analyser can work with either battery or mains A/C power. Fluorescently-labelled anti-CD3 and anti-CD4 antibodies are incubated with the sample, which once stained, is transferred to a detection channel where the signal is measured by the analyser. With a cost of $8.70 per test, this technology was designed for low-resource settings [42,43]. They also developed a system, called Alere Connectivity solution, to connect Pima analysers located in isolated areas to a centralised data warehouse, bringing the availability of high quality lab services to rural areas in developing countries. FDA-approved lab-on-a-disc platform Piccolo analysers introduced by Abaxis (see Figure 3) consist of a disposable, self-contained plastic disc and an analyser. The blood sample is loaded onto the disc, which is then placed into the analyser tray. Using centrifugal and capillary forces, plasma separation, metering, dilution, mixing, and distribution to the cuvettes containing the lyophilised reagents take place in the disc. The Piccolo has been used by the French, British, and US Military in remote locations and has also proved to be an appropriate blood chemistry analyser for biosafety environments, such as those required for monitoring Ebola patients [44]. Claros Diagnostics (now part of OPKO Health) is another company that has focused its efforts on the development of POC diagnostic devices for developing countries. Their platform consists of a blood-collector device, a disposable cartridge, and a reader, and obtained the CE Mark for the detection of prostate cancer-associated prostate specific antigen. This assay is based on a sandwich assay in which a gold nanoparticle-conjugated antibody is used in the detection process. The reduction of silver ions on these gold nanoparticles yields an amplified signal that can be optically measured. OPKO also developed a test for the detection of a panel of sexual-transmitted diseases (HIV, syphilis, and hepatitis C) in collaboration with Samuel K. Sia (co-funder of Claros Diagnostics) of Columbia University. A smartphone dongle (microfluidic cassette) was used to run the assays, which showed a sensitivity of 92%–100% and specificity of 79%–100% in a field study in Rwanda [45]. Iquum has a FDA-approved test for Flu A/B and have also developed a HIV test using the same analyser. Liat’s HIV Quant whole-blood assay was evaluated in South Africa yielding a specificity of approximately 88%. Further development of this test is still required [46].

The implementation of new POC diagnostic tools must overcome regulatory challenges. Many emerging microfluidic POC technologies have significant but unproved potential to improve global health. Regulatory processes, despite being time-consuming, are essential to assure the effectiveness and reliability of diagnostic tests. In locations where laboratory equipment and trained personal are not available, the use of diagnostic devices is regulated by the requirements of the Clinical Laboratory Improvement Amendments of 1988 (CLIA) in addition to the traditional Premarket Approval (PMA) or the approval via the 510(k) pathway in the U.S.A. For diagnostic tests to be CLIA-waived they must be simple and have a minimum risk for generating erroneous results. This means that sample preparation and fluid handling steps must be minimised, ideally by storing reagents on the microfluidic device and automatically releasing them. Chin *et al.* [7] summarised the CLIA-waiver requirements that must be taken into account when designing a diagnostic device intended to be used in developing countries. These are: (1) it should be a self-contained and automated test, allowing the use of unprocessed specimens; (2) it should not require technical training; (3) it should provide easily-interpreted results; and (4) it should be a robust test, able to handle variability in performing timings, storage conditions, *etc.* [7].

### 3.2. Self-Contained and Automated Testing

It is a challenge to integrate sample preparation and processing and detection techniques in a self-contained POC system. Non-instrumental, disposable-only tests would be ideal for delivering health care in low-resources settings as they are cheap and simple to perform. Thus, “disposable-only” microfluidic devices are especially suited for developing country diagnostics. Currently, these non-instrument-based tests are found in three different formats: lateral flow, flow-through, or solid phase. Unfortunately, the limitations in the limit of detection inherent to these platforms makes those disposables with an external reader a better alternative in many cases, in particular when the tested analyte is not very abundant in the sample [47].

The integration of multiple preparative and analytical microfluidic technologies in a single device allows the use of unprocessed samples to run an entire assay at a POC setting. Preconditioning of samples, when needed, is possible thanks to centrifugation, which allows the purification of plasma from blood, or microfluidic technologies such as an on-chip filter trench where blood cells are sedimented and captured [35,48]. As mentioned previously, microfluidic systems facilitate the miniaturisation of assays, thus reducing the surface-to-volume ratios, enhancing aspects of assay kinetics and allowing significant reductions in sample/reagent volumes. In this context of micrometre-sized channels, sample flow is laminar, and fluid mixing needs to be achieved by means other than traditional turbulent diffusion. Controlled fluid flow and mixing is essential for biosensing and in a microfluidic system this can be accomplished by passive methods (e.g., by lamination, use of specific surface chemistries for immobilisation of reagents, minimisation of non-specific binding, generation of hydrophobic/hydrophilic regions, and by use of specific geometries or topographies of channels), by the use of micro-pumps or micro-valves, or by applying an external energy supply [49]. The ability of sacrificial valves to create vapour barriers makes them ideal for reagent storage. Traditionally, sacrificial valves required external actuators (e.g., laser diodes) in order to be activated. The use of all external actuators except spindle motor can be excluded by using new valve designs, such as dissolvable-film (DF) valves that dissolve beyond a critical rotational frequency [50]. Since passive methods do not require external sources of energy, they are the most suitable for use in those areas with limited technological resources. Suitable alternatives are the aforementioned centrifugal microfluidic platforms, which offer precise fluid control and only require a simple motor for powering the system [38]. The technology needed to drive the microfluidic discs is as simple as that used in CD players or DVD drivers. Glynn *et al.* [51] developed a microfluidic “lab-on-a-disc” device that was able to purify cells expressing the HIV/AIDS-relevant epitope, CD4, from whole blood using centrifuge-magnetophoresis for cell isolation. This proved to be a cheap, simple, and efficient method that could be exploited for on-site diagnosis. Such microfluidic platforms can perform relatively complex analytical tests, which make them highly suitable for healthcare applications in remote areas.

### 3.3. No Specialised Training Requirements

**Figure 4:**
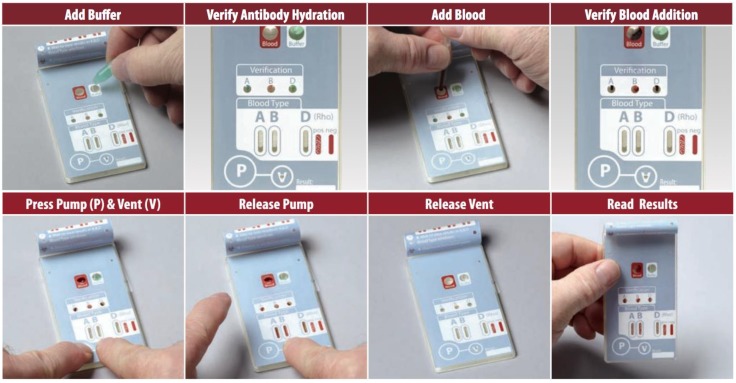
ABORhCard summarised assay procedure. Buffer is manually added by the user to re-hydrate the antibodies and this process is confirmed by the visualisation of colours in the A, B, and D “verification windows”. Then, using the lancet provided, a fingerstick blood sample is collected and added into the “blood” well. Once the colour appears in the “verification window”, the blood and reagents mix and flow into the “results window” using the on-board Pump (P) and Vent (V). At the end of the procedure, agglutination occurs and results can be visualised. Images taken from www.micronics.net with permission from Micronics.

Beyond the technical challenges, successful implementation of a diagnostic test in remote settings relies on the ability of the user to operate the test. The development of an appropriate interface is of utmost importance in those low-resource areas where healthcare workers are often not professional clinicians. These individuals would be able to perform basic tests, such as ICS strips for pregnancy or malaria, but may encounter difficulty if they need to prepare the samples or handle different reagents [35]. Microfluidic platforms overcome this problem by integrating sample preparation and all the immunoassay steps into a disposable self-integrated cartridge, which contains all the necessary reagents to run the test, allowing an automised analysis and minimising “hands-on” time. Once the sample is added to the cartridge, washing and calibration steps occur with no further liquid handling required. This reduces the exposure of the sample to the environment (avoiding contamination) and the operator-associated variability in the results. An example of a user-friendly test is the ABORhCard (from Sony) for the determination of the ABO group and Rh factor from a fingerstick blood sample. This device has been FDA-cleared and it is being used by the U.S. military in the Middle East, proving its suitability for austere environments. Anti-A, anti-B, and anti-D (Rh) antibodies are contained in channels in the card and re-hydrated by the addition of buffer prior to sample addition. The steps required to perform the test are summarised in Figure 4.

All of the steps involved in the diagnostic procedure should be simple and as culturally independent as possible. Reading a basic instruction manual written in non-technical language should enable the user to operate the test without any further “hands-on” training. The training requirements of a variety of FDA-approved microfluidics-based POC tests are shown in Table 2.

### 3.4. Easily Interpreted Results

A diagnostic test usually gives information regarding the presence/absence or concentration of the analyte of interest contained in a biological sample [47]. Healthcare workers in remote areas in developing countries may have little training, so it is desirable that the results are presented in a simple format to avoid misinterpretation. For example, Biosite (Alere) developed a system test with up to 16 immunoassays that provides qualitative results displayed as positive (POS) or negative (NEG) that are easy to interpret. Another alternative is transmitting the results data to a central laboratory with a cell phone for telemedicine, so experts can do a real-time analysis of the results. This was the chosen approach by Martinez *et al.* [52], who described a system in which they combined an inexpensive paper-based microfluidics diagnostic platform with a camera phone to quantify the results and exchange the information with trained staff. Smartphones are available worldwide, even in remote areas, and telemedicine offers a useful starting point for diagnostic platforms in low-resource areas.

One of the strengths of microfluidics is that they are able to produce sensitive results by detecting the signal produced in a very small area using fluorescence or other more recently implemented but ultrasensitive detection methods. Electrochemical detection, in particular, has been widely used for improving the sensitivity and also often the specificity of traditional tests, generating quantitative testing due to the use of portable electrochemical analysers in POC platforms. For example, the i-Stat blood gas analyser (see Table 2) combines self-contained cartridges with microfluidic components and electrochemical detection (including amperometry, voltammetry, and conductance, depending on the testing analyte). Each cartridge contains a sample chamber, a calibration solution, a waste chamber, and a unique combination of sensors, depending on the test. i-Stat analysers are battery-powered and have been used in some remote health centres in Australia [53], where, however, it is not yet part of the standard equipment provided. In developing countries, the main obstacle for the implementation of this test is the relatively high cost—an i-STAT analyser costs approximately $6000. Despite this, the price of each test (approximately $9) is still lower than performing the analysis in a central laboratory [54], and could be further reduced for implementation in developing countries.

### 3.5. Necessity of a Robust Test

For POC testing to reach its full potential in a low-resource setting, disposables need to be robust, allowing shipping and storage at ambient temperature. Reagents and antibodies for immunoassays must be stored on the disposable element. To avoid degradation, biomolecules would ideally be stored in a lyophilised state in vapour-proof compartments. This would protect their integrity, allowing them to be stored at ambient temperature and also would reduce the weight of disposables, which would contain only desiccated reagents. If the use of liquid reagents is necessary, they should be contained in a sterile and sealed environment to prevent evaporation [35]. Dry reagents enclosed in small cartridges or disposal discs that do not require to be refrigerated are best suited for resource-limited areas. That is the case of the Pima CD4 test, Piccolo^®^ reagent discs or the ABORhCard, already described in this review.

To test the efficiency of the test, a calibration check should be run in parallel to the test, ideally including both positive and negative controls. Thus, a microfluidic platform in which both a standard curve and the analyte test can be simultaneously generated on the same disc or chip is particularly attractive [27].

### 3.6. General Observations

Microfluidic technologies offer significant benefits for implementation in developing areas. Reducing the cost of materials to be used in the disposables is a must be taken into consideration. To minimise manufacturing costs, microfluidic devices can be made of plastic (produced by lamination, injection moulding, or embossing) or paper, both inexpensive materials appropriate for the fabrication of disposable devices [7]. In addition, lab-on-a-chip and lab-on-a-disc technologies have demonstrated their utility for use in remote areas, as they are compact, easy to use, and multifunctional. Individual components of microfluidic platforms, such as those needed for sample pre-treatment or reagent mixing have undergone significant improvements in recent years. Research efforts should now focus on the integration of all these components into a self-contained device. Only when this is achieved, and the systems become fully compliant with regulatory requirements, can such new clinically-relevant POC devices be brought to the market and their full potential exploited for improved global healthcare.

## 4. Conclusions

Developing countries have a much higher mortality rate from diseases such as HIV, TB, and malaria due to poor medical infrastructure in comparison to the developed countries. In addition to the communicable diseases, non-communicable diseases are also imposing a growing burden upon the resource-limited countries. The development of inexpensive, low-maintenance, reliable, and easy-to-use diagnostic tools can easily reduce the burden and impact of diseases in these environments. Despite the presence of high disease burdens and an ever-growing need, there are relatively few diagnostic tools that have been designed for fulfilling the specific needs and challenges of the developing countries. Currently, the only highly successful point-of-care (POC) testing platform available in the rural areas of developing countries is the lateral flow assay LFIA strip, which has contributed greatly to the identification of diseases without the requirement for any modern laboratory set-up. In spite of the advantages offered by LFIA strips, there are several inherent associated weaknesses, as indicated by clinicians and researchers, such as poor reproducibility and accuracy, and low sensitivity. Advancements in nanotechnology, microfluidics, and device fabrication have opened up new horizons in the field of diagnostic platforms for their successful placement in low-resource laboratory settings. Their intrinsic advantages, such as small size, manipulation of small volumes of liquids, high capability of integration, and rapid reactions offered by microfluidic technology make them promising candidates as POC diagnostic platforms. The ability of microfluidics based-platforms to accomplish all analytical steps such as sample pretreatment, assay, and detection, on a single easy-to-use, accurate and automatic test system makes them an ideal future diagnostic tool to provide real clinical value in low-resource, laboratory-free settings.
